# The conductance and organization of the TMC1-containing mechanotransducer channel complex in auditory hair cells

**DOI:** 10.1073/pnas.2210849119

**Published:** 2022-10-03

**Authors:** Robert Fettiplace, David N. Furness, Maryline Beurg

**Affiliations:** ^a^Department of Neuroscience, University of Wisconsin School of Medicine and Public Health, Madison, WI 53706;; ^b^School of Life Sciences, Keele University, Keele ST5 5BG, United Kingdom

**Keywords:** cochlea, TMC1, hair cell, LHFPL5, MET channel

## Abstract

Transmembrane channel-like protein 1 (TMC1) is thought to form the ion-conducting pore of the mechanoelectrical transducer (MET) channel in auditory hair cells. Using single-channel analysis and ionic permeability measurements, we characterized six missense mutations in the purported pore region of mouse TMC1. All mutations reduced the Ca^2+^ permeability of the MET channel, triggering hair cell apoptosis and deafness. In addition, *Tmc1* p.E520Q and *Tmc1* p.D528N reduced channel conductance, whereas *Tmc1* p.W554L and *Tmc1* p.D569N lowered channel expression without affecting the conductance. *Tmc1* p.M412K and *Tmc1* p.T416K reduced only the Ca^2+^ permeability. The consequences of these mutations endorse TMC1 as the pore of the MET channel. The accessory subunits, LHFPL5 and TMIE, are thought to be involved in targeting TMC1 to the tips of the stereocilia. We found sufficient expression of TMC1 in outer hair cells of *Lhfpl5* and *Tmie* knockout mice to determine the properties of the channels, which could still be gated by hair bundle displacement. Single-channel conductance was unaffected in *Lhfpl5*^−/−^ but was reduced in *Tmie^−/−^*, implying TMIE very likely contributes to the pore. Both the working range and half-saturation point of the residual MET current in *Lhfpl5*^−/−^ were substantially increased, suggesting that LHFPL5 is part of the mechanical coupling between the tip-link and the MET channel. Based on counts of numbers of stereocilia per bundle, we estimate that each PCDH15 and LHFPL5 monomer may contact two channels irrespective of location.

## Significance

We studied the role of TMC1 as the central component of the hair cell mechanotransducer (MET) channel by characterizing transduction in mice harboring mutations in the pore region. All *Tmc1* mutations reduced the Ca^2+^ influx into the hair bundle. Two mutations (*Tmc1 p.D528N* or *Tmc1 p.E520Q*) also decreased channel conductance and two (*Tmc1 p. D569N* or *Tmc1 p.W554L*) lowered expression. These mutations endorse TMC1 as the pore of the MET channel. The MET channel also contains accessory subunits, LHFPL5 and TMIE. MET currents were small in *Lhfpl5* or *Tmie* knockout mice. Nevertheless, MET channels could still be activated by hair bundle displacement; single-channel conductance was unaffected in *Lhfpl5*^−/−^ but reduced in *Tmie^−/−^,* suggesting TMIE likely contributes to the pore.

Hair cells of the inner ear convert acoustic and vestibular stimuli into electrical responses through activation of mechanically sensitive ion channels of incompletely defined organization. In the case of the auditory system, each cochlear hair cell converts sound-evoked vibrations of its stereociliary bundle into an electrical facsimile of the acoustic waveform. Each mechanoelectrical transducer (MET) channel is activated by tension in a tip-link extending from the side wall of one stereocilium to the tip of the adjacent shorter stereocilium (1, 2), where the channel is located (3). Over the last decade, a consensus has emerged that MET channels of cochlear hair cells comprise a pore-forming subunit, transmembrane channel-like protein 1 (TMC1) (4–8), aided by three accessory proteins, LHFPL5 (9, 10), TMIE (11–13), and CIB2 (14–16). Force is applied to the channel by protocadherin 15 (PCDH15), homodimers, which create the lower end of the tip-link (17, 18). However, the channel structure and the contributions of the accessory subunits are uncertain, and the final route, whereby mechanical stimuli activate the channel protein, is unknown. The structure of the mammalian MET channel has not yet been described. However, a recent cryoelectron microscopy (cryo-EM) study of the TMC1 complex in *Caenorhabditis elegans* indicated an assembly of two copies each of the pore-forming TMC1 subunit, the transmembrane (TM) inner ear protein TMIE, and the calcium-binding protein CALM-1 homologous with CIB2 (19). The location of TMIE in the structure indicates that it may contribute to the pore as previously suggested (13).

Nevertheless, multiple functional questions need addressing. For example, the current model of TMC1 (7, 20), based on its similarity to the Ca^2+^-activated Cl^−^ channel TMEM16, has a large extent of the pore exposed to the membrane lipid. This feature is related to the purported phospholipid scramblase activity of TMEM16 as a template for the TMC1 model (20). How can this exposed pore region be reconciled with the tight cationic selectivity of the channel, particularly its high discrimination for Ca^2+^ ions? A related paradox arises in allying a modest single-channel conductance (21, 22) with the large pore diameter of 1.25 nm estimated from channel permeability to organic cations (23) and aminoglycoside antibiotics (24). A second problem concerns the origin of the tonotopic gradient in the TMC1 channel conductance (22, 25, 26). Does this twofold gradient stem from modulation of the pore and why is it absent with the TMC2 isoform (21, 22), which dominates transduction during early cochlear development in mice (6, 22)? Third, do the accessory subunits LHFPL5 and TMIE contribute to the TMC1 channel pore or are they required solely to target TMC1 to the transduction site at the tips of the shorter stereocilia?

The aim of this article is to address these questions using evidence from our recent work on the effects of mutations of *Tmc1*, *Lhfpl5*, and *Tmie* on single-channel properties. Single MET-channels were characterized by recording currents in whole-cell mode after brief exposure of the bundle to saline containing submicromolar Ca^2+^ buffered with BAPTA [(1,2-bis(*o*-aminophenoxy) ethane-N,N,N′,N′-tetra-acetic acid)]. This procedure severs most of the tip-links that transmit force to the MET channel, leaving one or two channels activatable by bundle deflection. We first used this method while studying transduction in turtle auditory hair cells (27), and subsequently applied it to mammalian cochlear hair cells (22, 26).

## Methods

### Mouse Mutants.

*Tmc1* p.D528N, *Tmc1* p.D569N, *Tmc1* p.W554L, *Tmc1* p.T416K, and *Tmc1* p.E520Q mice were made by Horizon Sage Labs Inc. using CRISPR/Cas9 technology. *Tmc1* p.M412K (*Beethoven*) mice were obtained from Karen Steel, Kings College, London, United Kingdom, and Walter Marcotti, Sheffield University, United Kingdom. The *Tmie^−/−^* (transmembrane inner ear) mouse strain was B6.B(CBA)*-Tmie^sr^*/J (Jackson Labs, strain 000543). The *Lhfpl5^−/−^* (lipoma MMGIC fusion partner-like 5) mouse strain was the B6.129-*Lhfpl5^tm1Kjn^*/Kjn (Jackson Labs, strain 005434). All mutants used for characterizing hair cell transduction were bred on a *Tmc2^−/−^* background to circumvent complications due to different channel properties of TMC2 (5, 21). *Tmc2^−/−^* mice (B6.129S5-*Tmc2^tm1Lex^*/Mmucd) were obtained from the Mutant Mouse Regional Resource Center (University of California, Davis). Neonatal mice up to postnatal day (P)8 were killed by decapitation according to the animal protocol approved by the Institutional Animal Care and Use Committee at the University of Wisconsin–Madison.

### Hair Cell Recording and Stimulation.

MET currents were recorded from inner hair cells (IHCs) and outer hair cells (OHCs) in isolated organs of Corti of mice between P2 to P8, as previously documented (21, 28). The cochlear location of hair-cell recordings is specified by *d*, the distance along the basilar membrane from the apex divided by the total length of the basilar membrane, which in P6 mice has a mean of 5.2 mm. Recordings were made at an apical low-frequency position, *d* = 0.2 to 0.3, and a basal high-frequency position, *d* = 0.8. Since the magnitude of the MET current varies with OHC position along the cochlea, it is essential that mutant and control properties be compared at the same cochlear location. The recording chamber was mounted on the stage of a Leica DMLFS top-focusing microscope and viewed with 40× (NA = 0.8) objective and a 2× optivar. The chamber was perfused with saline: 152 mM NaCl, 6 mM KCl, 1.5 mM CaCl_2_, 2 mM Na-pyruvate, 8 mM d-glucose, and 10 mM Na-Hepes, pH 7.4. Patch electrodes were filled with : 138 mM CsCl, 3.5 mM MgCl_2_, 5 mM Na_2_ATP, 0.5 mM Na_2_GTP, 10 mM Tris phosphocreatine, 1 mM BAPTA, 10 mM Cs-Hepes, pH 7.2, and were connected to an Axopatch 200B amplifier. Electrode series resistances with 60% compensation were at best 3 MΩ, which, with a ∼5 pF cell capacitance, gave a recording time constant of 15 µs. All MET currents were smoothed with a Frequency Device LPO3 8-pole filter at 3 kHz. Stereociliary bundles were stimulated with a fluid jet or with a glass probe driven by a piezoactuator. For both types of stimulator, the amplitude of motion was calibrated by projecting the bundle image onto a pair of photodiodes and measuring the change in photocurrent (29, 30). The relationship between the MET current, *I*, scaled to its maximum value *I*_MAX_, and bundle displacement *X* was fitted with a single Boltzmann equation *I*/*I*_MAX_ = 1/(1 + exp((*X*_O_ − *X*)/*X*_S_), where *X*_O_ is the half-saturating displacement and *X*_S_ is a slope factor; the 10 to 90% working range (WR) is 4.4 × *X*_S_. According to the gating spring model, *X*_S_. = *k*_B_*T*/*Z*, where *k*_B_ is the Boltzmann constant and *Z* is the gating sensitivity (31). *I–X* relationships were determined from the first cycle of response to sinusoidal fluid jet stimulation. The Ca^2+^ selectivity of the MET channel relative to Cs^+^ was determined from Ca^2+^ reversal potentials of the MET current measured in an extracellular solution containing 100 mM CaCl_2_, 20 mM *N*-methylglucamine, 5 mM Tris, pH 7.4, and an intracellular Cs-based solution. Reversal potentials were corrected for a −9-mV junction potential and were analyzed using the Goldman-Hodgkin Katz equation with activity corrections applied to the ion concentrations (5, 26). Experiments were performed at room temperature, ∼23 °C. Results are quoted as means ±1 SD and statistical significance was assessed using a two-tailed Student *t* test.

### Single-Channel Conductance.

Single MET-channel currents were recorded in whole-cell mode after brief exposure of the bundle to saline containing 5 mM BAPTA plus 1.0 mM Ca^2+^ (22, 26). Treatment with submicromolar Ca^2+^ buffered with BAPTA severs most tip-links transmitting force to the MET channel, leaving one or two channels that can be activated by bundle deflection. It is unlikely that BAPTA exposure alters channel conductance since identical channel amplitudes could be obtained in the absence of BAPTA in 2-d-old postnatal mice during early development of the MET current (32). The most serious problem encountered is that single-channel events may be difficult to isolate because of fast current transitions causing multiple current levels to be merged into one, with the result that the unitary conductance is overestimated. It was therefore important to obtain long stretches of channel records to search for smaller events. Nonstationary fluctuation analysis of the macroscopic MET current, which has occasionally been used to derive single MET channel amplitudes (33), substantially underestimates channel size because of filtering of the current by the recording system (32) and so cannot provide a quantitative measure of channel conductance. We sometimes observed in the single-channel recordings a subconductance level of ∼40 pS, but here we focused on the most prevalent state with conductance of 70 to 80 pS in apical OHCs (22). An infrequent subconductance level about 40% of the dominant level was originally seen in turtle auditory hair cells (25, 27).

### Scanning Electron Microscopy.

Hair bundle morphology and stereociliary counts were performed on scanning electron micrographs (SEM) of cochleae of 30-d-old CD1 mice. Maintenance and treatment of mice during preparation was in accord with the UK Animals (Scientific Procedures) Act of 1986. P30 mice were anesthetized with sodium pentobarbitone, decapitated, the cochleae removed and fixed through holes in the round window, and the cochlear apex with 2.5% glutaraldehyde (GTA) in 0.1 sodium cacodylate buffer plus 2 mM CaCl_2_ for 2 h. Dissected spirals were postfixed in 1% OsO_4_/sodium cacodylate buffer and then impregnated with osmium-thiocarbohydrazide using the OTOTO technique (34). Cochleae were dehydrated through an ethanol series, critical point dried from liquid CO_2_, mounted onto SEM stubs, and examined in a Hitachi S4500 field emission SEM at 5 kV.

## Results

### TMC1 and TMC2.

The main pore-forming component of the MET channel in cochlear hair cells is thought to be TMC1 (7). TMC1 was identified by positional cloning of a gene underlying nonsyndromic sensorineural hearing loss (35) and was shown to be one of two isoforms, along with TMC2, that is required for mechanoelectrical-transduction in hair cells of the mouse inner ear (6). In the mouse cochlea, the TMC2 isoform was found to be expressed early in development but was subsequently replaced by TMC1 in the adult (5, 6, 22). For a period between P2 and P8, when most recordings from mouse cochlear hair cells are made, both TMC1 and TMC2 are present. This poses problems in documenting MET channel properties because the two isoforms create channels differing in their unitary conductance (21), Ca^2+^ permeability (5), adaptation (36), and interactions with accessory subunits (10). It is therefore essential that characterization of TMC1-containing MET channels during this period be done on mice on a *Tmc2* knockout background. *Tmc2^−/−^* has no auditory phenotype and, moreover, TMC2 cannot fully substitute for TMC1 in the adult (37). Modeling of TMC1 based on the related Ca^2+^-activated chloride channel TMEM16A (38), the structure of which is known from cryo-EM (39), has suggested that the TMC1 channel is dimeric. Each monomer is comprised of 10 transmembrane (TM) domains (TM1 to TM10), with the ion-conducting pore placed between TM4 and TM7 (7, 19, 20).

### Three Functional Consequences of *Tmc1* Mutations.

**Fig. 1.**
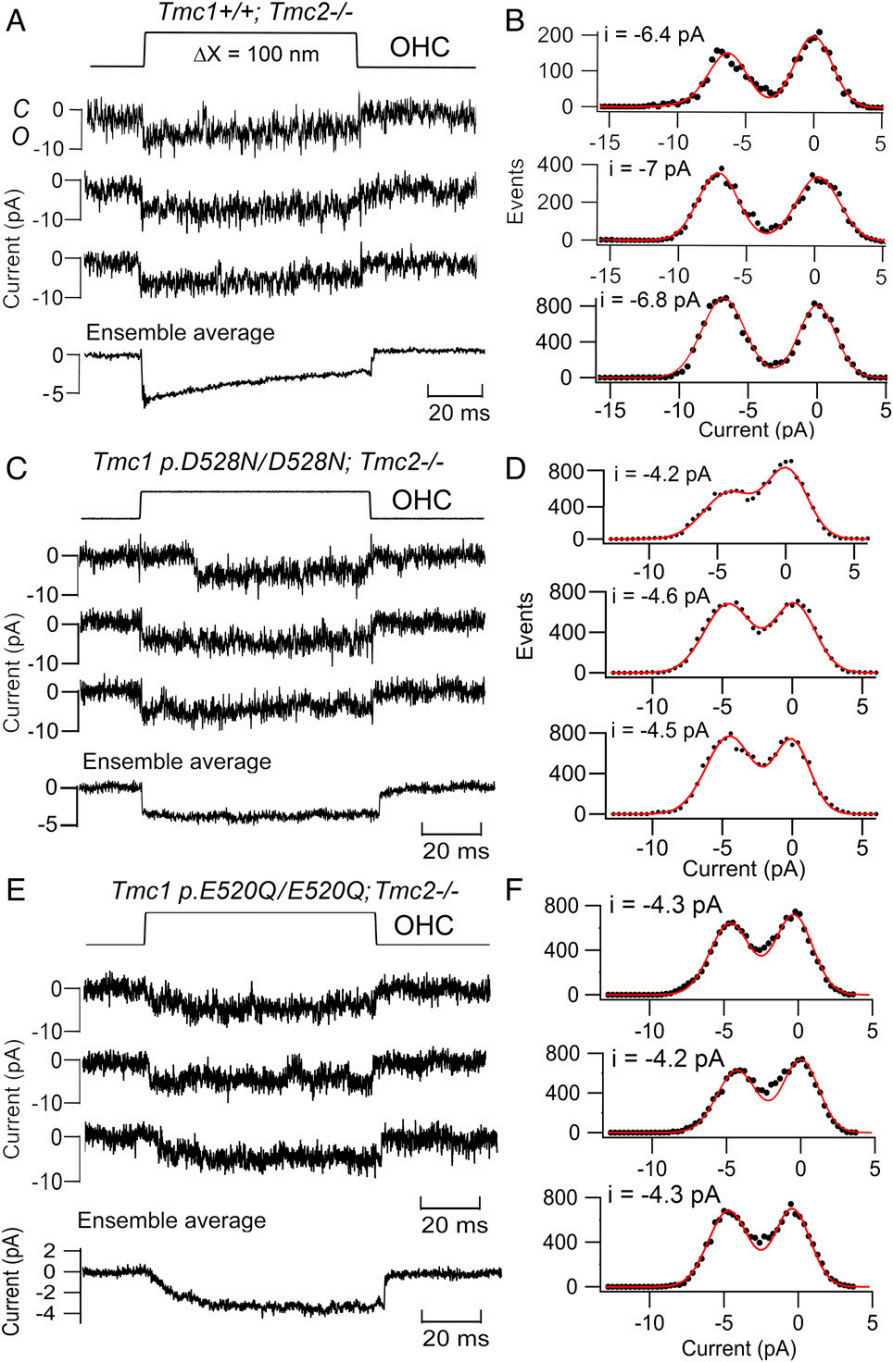
*Tmc1* mutations that reduce MET channel conductance in OHCs. (*A*) Control *Tmc1^+/+^;Tmc2^−/−^* single-channel currents in response to a step displacement (ΔX, *Upper*) of the hair bundle, ensemble average of 50 stimuli below. (*B*) Amplitude histograms of records on left, mean −6.7 ± 0.3 pA. (*C*) *Tmc1* D528N/D528N*;Tmc2^−/−^* single-channel currents for 100-nm bundle displacement, ensemble average of 30 stimuli below. (*D*) Amplitude histograms of records on left, mean −4.4 ± 0.3 pA. (*E*) *Tmc1* E520Q/E520Q*;Tmc2^−/−^* single-channel currents for 100-nm bundle displacement, ensemble average of 60 stimuli below. (*F*) Amplitude histograms of records on left, mean −4.3 ± 0.3 pA. Both mutations produce ∼35% amplitude reduction. Holding potential −84 mV; all OHCs located at *d* = 0.3 in P5 mice.

Our studies of hair-cell MET channels in mice harboring mutations of *Tmc1* in the putative pore region revealed three functional consequences: changes in conductance, expression, and Ca^2+^ permeability. Neutralizing a negative aspartate, *Tmc1* p.D528N, in TM6 near the external face of the membrane, reduced the channel conductance in apical OHCs from 81 ± 3 pS (*n* = 5 cells; *Tmc1^+/+^;Tmc2^−/−^*) to 53 ± 3 pS (*n* = 5 cells; *Tmc1* p.D528N/D528N*;Tmc2^−/−^*) (Fig. 1 *A*–*D*). The smaller conductance was accompanied by a much decreased selectivity for Ca^2+^ ions: *P*_Ca_/*P*_Cs_, (ratio of permeability to Ca^2+^ ions relative to Cs^+^ ions) = 4.3 ± 0.4 (*n* = 8) in *Tmc1^+/+^;Tmc2^−/−^* and 0.6 ± 0.04 (*n* = 7) in *Tmc1* p.D528N/D528N*;Tmc2^−/−^* (4). These results demonstrate that the D528 residue affects ion transport into the pore, and neutralizing that charge reduces access to monovalent cations and Ca^2+^ ions. A similar reduction in channel conductance was achieved by neutralizing an adjacent acidic residue: the mutation *Tmc1* p.E520Q reduced the channel conductance to 52 ± 4 pS (average of 75 traces in 4 cells) (Fig. 1 *E* and *F*). There was also decreased selectivity for Ca^2+^ ions: *P*_Ca_/*P*_Cs_, (ratio of permeability to Ca^2+^ ions relative to Cs^+^ ions) = 2.0 ± 0.4 (*n* = 10) in *Tmc1* p.E520Q/E520Q*;Tmc2^−/−^*. The similar effects of D528N and E520Q on channel conductance are consistent with these residues being in the external neck of the pore to restrict the influx of ions. The *Tmc1* p.D528N/D528N mutation also drastically increased (by 67-fold) the half-blocking concentration of the aminoglycoside antibiotic dihydrostreptomycin (i.e., decreased the blocking sensitvity), suggesting a tight electrostatic control of access to the pore.

**Fig. 2.**
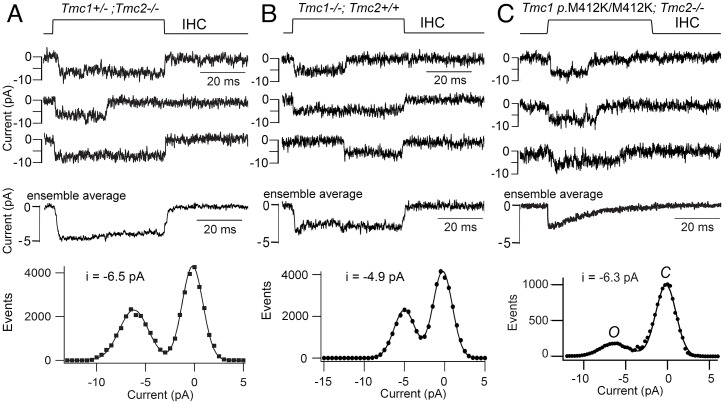
MET channel currents in IHCs from *Tmc1* mutant mice. (*A*) *Tmc1^+/−^;Tmc2^−/−^* single-channel currents for bundle displacements (*Top*) and ensemble average for 100 stimuli (*Middle*). Amplitude histogram (*Bottom*) gives single-channel current of −6.5 pA. (*B*) *Tmc1^−/−^;Tmc2^+/+^* single-channel currents for bundle displacements (*Top*) and ensemble average for 50 stimuli (*Middle*). Amplitude histogram (*Bottom*) gives single-channel current of −4.9 pA, indicating that TMC2 channels have smaller current amplitudes than TMC1 in *A*. (*C*) *Tmc1* M412K/M412K*;Tmc2^−/−^* single-channel currents for bundle displacements (*Top*) and ensemble average for 100 stimuli (*Middle*; taken from ref. 41). Amplitude histogram (*Bottom*) gives a single-channel current of *Tmc1* M412K/M412K (*Beethoven*) as −6.3 pA, no different from control in *A*. Holding potential: −84 mV. All IHCs were located at *d* = 0.2 in P4 to P5 mice.

Two mutations, *Tmc1* p.D569N and *Tmc1* p.W554L, which are located at the intracellular interface of TM7, reduced TMC1 expression with no alteration in single-channel conductance (4, 28). Evidence for reduced TMC1 expression in both mutants was a two-thirds reduction in both the maximum MET current and in TMC1 immunolabeling in the stereocilia. This effect has been hypothesized to result from a reduced interaction between TMC1 and the LHFPL5 subunit (40), which is required for transport of the MET channel complex to the shorter stereocilia at the lower end of the tip-link attachment point (10). The third set of mutations were *Tmc1* p.M412K and *Tmc1* p.T416K in TM4. While neither altered channel expression or single MET channel conductance, both reduced the selectivity of the channel for Ca^2+^ (4, 41, 42). The single-channel conductance in apical OHCs (4) was 84.8 ± 3.1 pS in control (*n* = 5 cells; *Tmc1^+/+^;Tmc2^−/−^*), 86.2 ± 8.2 pS (*n* = 5; *Tmc1 p.*M412K/M412K*;Tmc2^−/−^*), and 87.2 ± 4.5 (*n* = 5; *Tmc1 p.*T416K/T416K*;Tmc2^−/−^*); the value for neither mutant was significantly different from control (4). Both mutations cause dominant human deafness, as does the neighboring *Tmc1* p.G411R (using amino acid numbering in mouse) (43). These effects suggest there is a string of residues in TM4 that, when mutated to a positive lysine or arginine, cause deafness (4). We also found no difference in the IHC single-channel current amplitude between T*mc1 p.*M412K [often referred to as *Beethoven* (44)] and *Tmc1^+/+^;Tmc2^−/−^* (41), in contrast to the smaller channel current in the *Tmc1^−/−^;Tmc2^+/+^* (Fig. 2); the latter observation indicates that TMC1-containing channels have a third larger conductance than TMC2-containining channels.

There is disagreement about the effects of the *Tmc1* p.M412K mutation on transduction, and the mutation has been claimed to reduce single-channel conductance (8). The MET channel conductance reported by that group was 148 ± 90 pS in *Tmc1*^+/−^ and 102 ± 70 pS in *Tmc1* p.M412K/– (8). The discrepancy with our data may be partly attributable to their method of isolating channels, not using BAPTA but attempting to restrict mechanical stimulation to one column of stereocilia, thus recruiting single stereocilia from row 1, row 2, or row 3 (8). However, since the stereocilia are tightly coupled across the bundle, the events recorded in whole-cell mode may comprise multiple channels. An overestimate of the channel conductance was also evident in recordings of current fluctuations or noise (7), where it was reported that IHCs had a single-channel current of −13 pA (at −84 mV) compared to our value of −3.9 pA from measurements of current noise (32) and −6.5 pA when observing single-channel events (Fig. 2), both at −84-mV holding potential. The smaller amplitude of the MET channel current when derived from noise measurements is a necessary consequence of filtering by the limited time constant of the recording system, and allowed us to estimate the activation time constant of the MET channel as 10 µs (32).

Errors in the determination of single-channel conductances using noise analysis raise concerns with the significance of conductance changes induced by genetic or chemical manipulations (45). Methodological problems have troubled other studies on IHCs, even when isolation with BAPTA treatment was employed. For example, the first recordings of MET channels in rat IHCs gave a conductance of ∼180 pS (26), but it seems likely that this value reflected superposition of at least two channel events as the apical OHC conductance was 98 pS (26), not too different from mice. To unequivocally resolve the disagreement might require developing methods of recording from cell-attached patches containing just one channel on single stereocilia, a method typically employed to record other types of ion channel.

### Tonotopic Gradient in MET Channel Conductance.

**Fig. 3.**
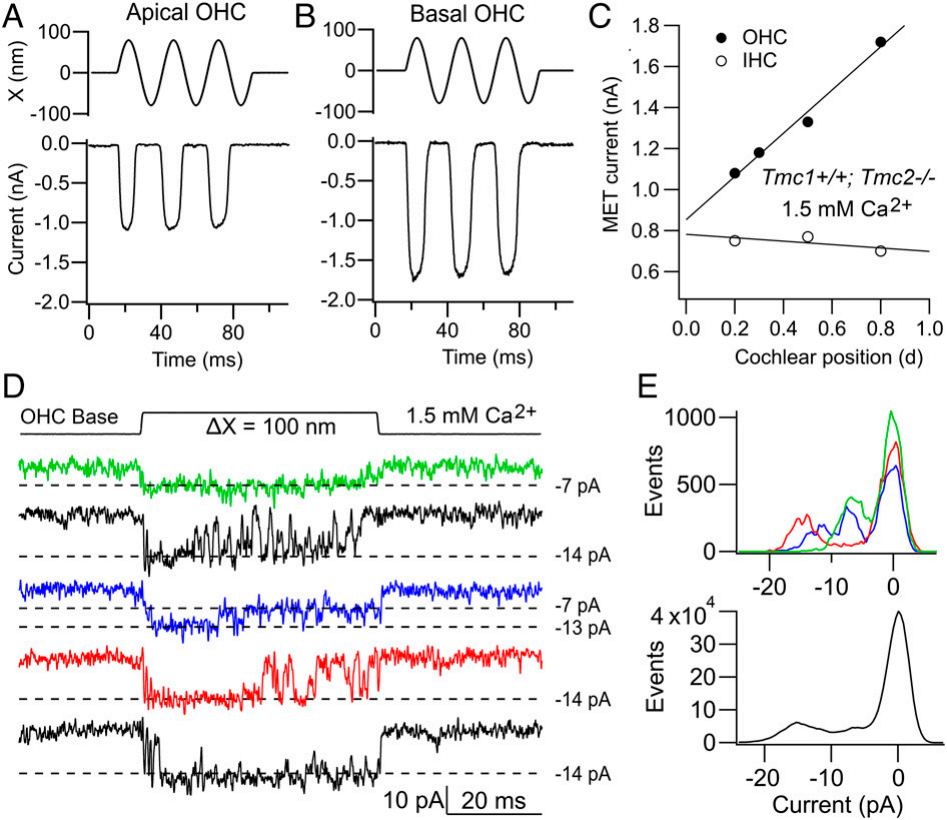
Tonotopic gradient in MET currents. (*A*) *Tmc1^+/+^;Tmc2^−/−^* apical OHC current for bundle displacements (*Upper*), OHC at *d* = 0.2. (*B*) *Tmc1^+/+^;Tmc2^−/−^* basal OHC currents for bundle displacements (*Upper*); OHC at *d* = 0.8. Holding potentials: −84 mV. (*C*) Variation in maximum MET current versus fractional distance along basilar membrane from apex for OHCs (filled circles) and IHCs (open circles) from *Tmc1^+/+^;Tmc2^−/−^* mice. Hair bundles stimulated with a 40-Hz fluid jet set to achieve a saturating current. The largest currents obtained at each location are plotted. (*D*) *Tmc1^+/+^;Tmc2^−/−^* single-channel currents in response to a step displacement (ΔX, *Top*) of the hair bundle for a basal OHC. Note presence of −14-pA and −7-pA channels. (*E*, *Upper*) Amplitude histograms of individual channel records in *D* showing peaks at −7 pA, −11 pA, and −14 pA; (*Lower*) amplitude histograms of all 50 channel records showing peak at about −15-pA at −84-mV holding potential. Results in *D* and *E* are taken from Beurg et al. (22).

MET currents from OHCs at the base of the cochlea, where high-frequency sounds are detected, are up to twice as large in amplitude as those at the low-frequency apex (Fig. 3 *A–C*). In contrast, the MET current gradient is negligible in TMC1-containing IHCs (Fig. 3*C*). MET channels in adult IHCs (as in OHCs) are most likely composed solely of the TMC1 isoform, a conclusion endorsed by the observation that IHC MET currents of *Tmc1^−/−^* mice vanish after P12 (22). The molecular origin of the conductance gradient is not fully understood, nor is it clear why it is absent in IHCs. Functionally, its presence in OHCs will increase the sensitivity of high-frequency cells augmenting their motor function (46). A possible mechanism for the tonotopic variation is a gradient in the single-channel current, which in many recordings appears larger at the base than at the apex in OHCs (22, 25, 26) (Fig. 3*D*). But are the large channels distinct or do they stem from superposition of two smaller channels? Discriminating discontinuities or steps in the onset of a channel event, hinting at multiple channels, can be difficult, making interpretation of changes in channel conductance problematic as was discussed above. In some recordings, a small fraction of the traces acquired display two distinct main conductance levels. Thus, for the basal hair cell (Fig. 3*D*), the most frequently observed level was ∼14 pA, but in a few traces, prolonged openings to 7 pA or transitions from 14 to 7 pA later in the response were seen. The 7-pA level was the most prominent level in apical OHCs during these recordings. The simplest conclusion is that the 14-pA level arises by summation of two 7-pA current levels. Amplitude histograms of a few traces show both conductance levels (Fig. 3 *E*, *Upper*), but histograms of many traces from this experiment exhibit predominantly an ∼14-pA level (Fig. 3 *E*, *Lower*).

The notion of generating large-amplitude channels using multiple channels of smaller conductance is consistent with analysis of MET current noise, which yields almost identical single-channel current amplitudes at both apex and base (32). This observation argues that channel current size is identical at both ends of the cochlea and that channel conductance increases along the cochlea by summation of multiple channels of the same size. The explanation avoids postulating a mechanism for modulating channel current amplitude. A drawback of producing a larger apparent conductance by adding extra small channels is that it is not obvious how each tip-link interacts with a variable number of TMC1 proteins according to cochlear location. Supernumerary channels, those not directly attached to the tip-link, could conceivably be gated by elastic forces arising via the lipid bilayer and inducing cooperative opening and closing of the channels (47). However, the possibility of different channel sizes cannot be excluded. There is recent evidence of an alternatively spliced version of TMC1 with deletion of residues 517 to 519 (VCQ) (48), immediately preceding the E520 that influences channel conductance. It is possible that this TMC1 variant confers a larger channel conductance, and interestingly it is expressed more highly at the base.

### Role of the Accessory Protein TMIE.

**Fig. 4.**
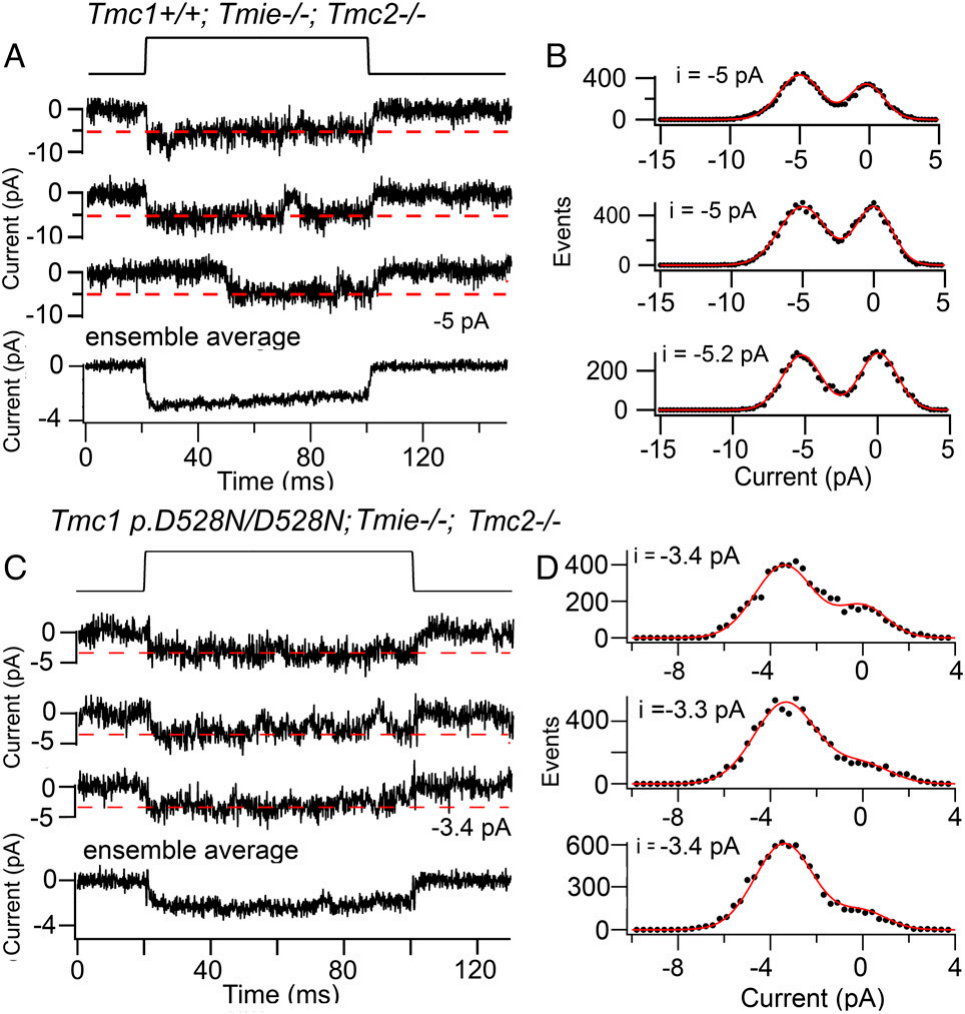
Single MET channel currents in apical OHCs of *Tmie* knockouts. (*A*) *Tmc1^+/+^;Tmie^−/−^* single-channel currents in response to a 100-nm step displacement of the hair bundle (*Top*), ensemble average of 50 stimuli shown below. (*B*) Amplitude histograms of records on left, mean −5.0 pA. (*C*) *Tmc1* D528N/D528N*;Tmie^−/−^* single-channel currents for 100-nm bundle displacement, ensemble average of 30 stimuli below. (*D*) Amplitude histograms of records on left, give mean −3.4 pA; holding potential −84 mV.

There are three known accessory subunits: TMIE, LHFPL5, and CIB2. TMC1 is thought to be absent from the tips of the stereocilia in *Tmie^−/−^* (12, 13) and in *Lhfpl5^−/−^* mice (10), suggesting that both subunits are necessary for transport and targeting of TMC1 to the transduction site. Do either TMIE or LHFPL5 contribute to the channel pore? We examined MET currents in OHCs of *Tmie* knockouts and observed the presence of small macroscopic currents (−30 ± 11 pA; *n* = 4), encouraging us to search for single channels (Fig. 4 *A* and *B*). A unitary current of −5.3 ± 0.4 pA (at −84 mV), was obtained from 60 traces in four apical OHCs of *Tmc1^+/+^;Tmie^−/−^;Tmc2^−/−^*, equivalent to a conductance of 63 ± 5 pS; this value is smaller than the control value of 85 ± 3 pS (*t* test, *P* = 0.001) in *Tmc1^+/+^;Tmie^+/+^;Tmc2^−/−^* measured at the same location. To confirm that we were indeed studying TMC1-containing MET channels, we crossed *Tmie^−/−^* with *Tmc1* p.D528N/D528N mice, which we had already found to reduce the TMC1 channel conductance. MET currents in apical OHCs recorded from *Tmc1* p.D528N/D528N*; Tmie^−/−^; Tmc2^−/−^* had a reduced size of −3.3 ± 0.4 pA (*n* = 4 OHCs). The ratio of the MET channel current in *Tmc1* p.D528N/D528N*; Tmie^−/−^;Tmc2^−/−^* relative to *Tmc1^+/+^;Tmie^−/−^;Tmc2^−/−^* was 0.63, identical to the ratio of channel current amplitudes of *Tmc1* p.D528N/D528N*;Tmie^+/+^;Tmc2^−/−^* to *Tmc1^+/+^;Tmie^+/+^;Tmc2^−/−^*, which was also 0.63. From these results, we conclude that MET channels can still be gated in the absence of TMIE, but they have a 25% smaller amplitude. A 20% reduction in channel amplitude from 58 pS to 46 pS was reported with the deafness-linked mutation *Tmie* p.R82C/R82C (13) on a *Tmc1^−/−^Tmc2^+/+^* background, where the wild-type channel conductance is smaller (Fig. 2). For TMC1-containing channels lacking TMIE, a pore across the membrane must exist to conduct ions and can still be opened by bundle deflection. However, the reduced channel amplitude suggests that TMIE may contribute to the pore. It should be noted that MET current events were rarely seen in *Tmie* knockouts, suggesting that TMIE, apart from being involved in ferrying TMC1 to the transduction complex, may also stabilize that complex. Consequently, *Tmie*-null mutants are deaf in mice (11) and lack microphonic potentials in zebrafish (49).

### Role of LHFPL5.

**Fig. 5.**
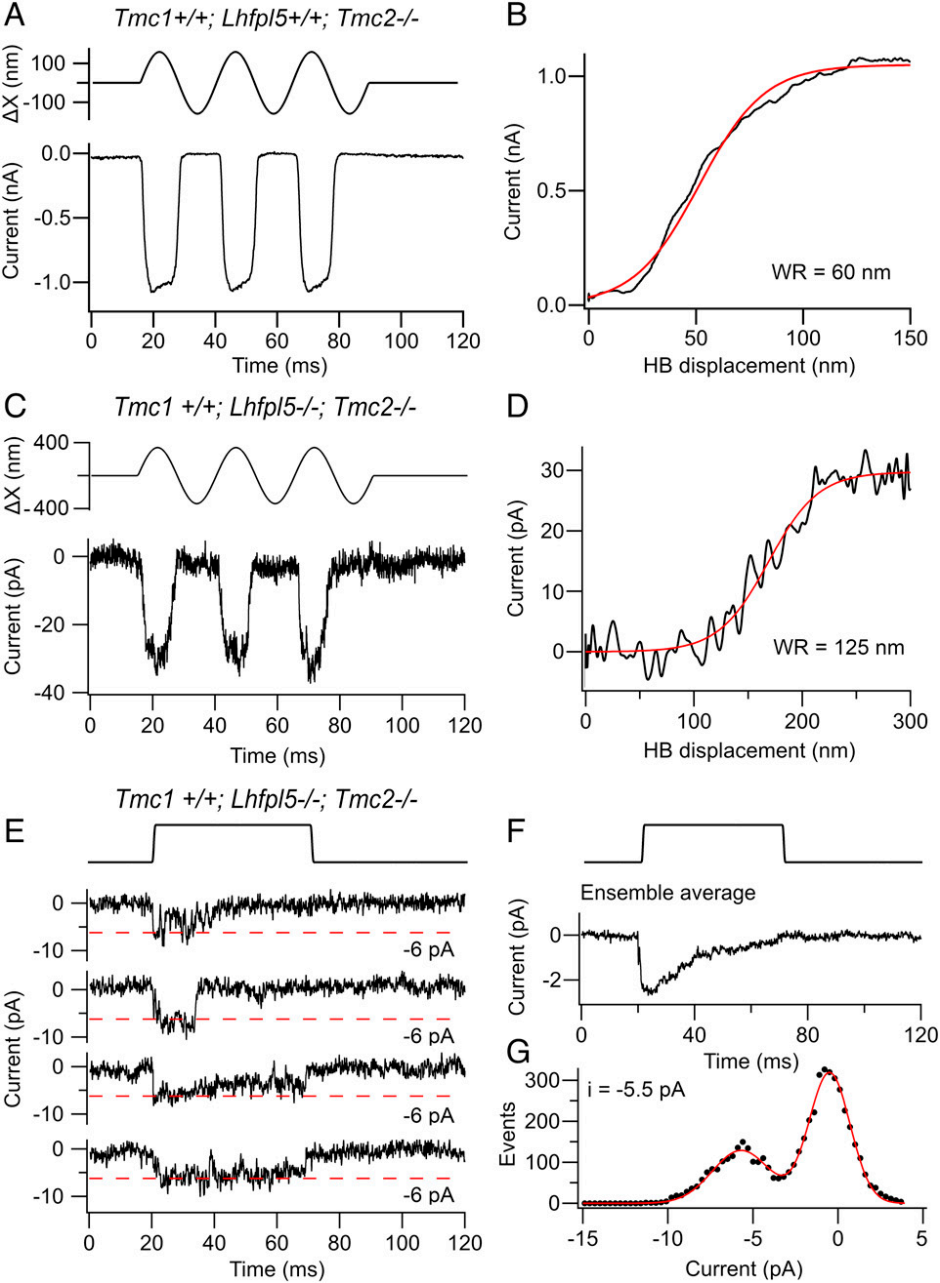
MET currents in OHCs of *Lhfpl5* knockouts. (*A*) Macroscopic currents for sinusoidal deflections of hair bundle in control *Tmc1^+/+^;Lhfpl5^+/+^;Tmc2^−/−^* mouse. (*B*) Current–displacement relationship from records in *A* fitted with a single Boltzmann with a 10 to 90% WR of 60 nm. (*C*) Macroscopic current for sinusoidal deflections of hair bundle in *Tmc1^+/+^;Lhfpl5^−/−^;Tmc2^−/−^*. (*D*) Current–displacement relationship from records in *C* fitted with a single Boltzmann with a 10 to 90% WR of 125 nm. (*E*) *S*ingle-channel currents in a *Tmc1^+/+^;Lhfpl5^−/−^;Tmc2^−/−^* OHC for 100-nm bundle displacement. (*F*) Ensemble average of 100 stimuli below. (*G*) Amplitude histograms of combined records on left, mean −6.0 pA. All measurements at −84-mV holding potential on OHCs located at *d* = 0.2 in P5 mouse.

We also examined the contribution of LHFPL5 to channel structure and found small MET currents in OHCs of *Tmc1^+/+^;Lhfpl5^−/−^;Tmc2^−/−^* mice compared to *Tmc1^+/+^;Lhfpl5^+/+^;Tmc2^−/−^* mice of similar age (Fig. 5 *A*–*D*). It was important to make measurements in *Tmc2* knockouts because this isoform does not require LHFPL5 for channel operation but still supports large MET currents in *Lhfpl5^−/−^* (10). Although MET currents have been previously reported in *Lhfpl5^−/−^* (9), they most likely flowed through channels containing solely TMC2, which may account for the smaller channel conductance reported and the slowed adaptation kinetics (36). In the absence of TMC2, apical OHCs generated MET currents (Fig. 5*C*) of 28 ± 6 pA (*n* = 5 OHCs), but these were 30-fold smaller than the maximum currents of 1,000 pA in *Tmc1^+/+^;Lhfpl5^+/+^;Tmc2^−/−^* of similar age (Fig. 5*A*). Nevertheless, the MET current response was reproducible and produced from channels of amplitude comparable to those in wild-type (Fig. 5 *E*–*G*). The mean single-channel current was −6.3 ± 0.8 pA (*n* = 51 channel events in 2 OHCs) at −84-mV holding potential, equivalent to a conductance 75 ± 11 pS. Control recordings in *Tmc1^+/+^;Lhfpl5^+/+^;Tmc2^−/−^* mice made at the same cochlear location (*d* = 0.2) gave a unitary current of 6.8 ± 0.5 pA (120 traces in 5 OHCs). There was no difference between the *Lhfpl5* knockout and control (*t* test, *P* = 0.13). Thus, as with *Tmie* knockouts, MET channels without LHFPL5 still possess an ion conduction pathway across the membrane that can be opened by hair bundle deflection; such channels also displayed marked adaptation (Fig. 5*F*).

Similar to *Tmie* knockouts, macroscopic currents were small in the mutant, implying that loss of either accessory subunit destabilizes the transduction channel complex. In addition to the much smaller MET current for the *Tmc1^+/+^;Lhfpl5^−/−^;Tmc2^−/−^*, the WR was about twice that in the *Tmc1^+/+^;Lhfpl5^+/+^;Tmc2^−/−^* (Fig. 5 *B* and *D*). When the current-displacement curves were fitted with a single Boltzmann equation, the 10 to 90% WR was 125 ± 18 nm in four *Tmc1^+/+^;Lhfpl5^−/−^;Tmc2^−/−^* mice, significantly larger (*t* test, *P* = 0.005) than the value of 70 ± 8 nm in four *Tmc1^+/+^;Lhfpl5^+/+^;Tmc2^−/−^*mice. The half-saturation *X*_O_ was also significantly increased, 153 ± 13 nm in four *Tmc1^+/+^;Lhfpl5^−/−^;Tmc2^−/−^* mice compared to 58 ± 8 nm in four *Tmc1^+/+^;Lhfpl5^+/+^;Tmc2^−/−^* mice. One explanation for the difference is that the mechanical connection from the tip-link to the MET channel was weakened in the absence of LHFPL5, thereby reducing the gating sensitivity or single-channel gating force (31). The delayed activation of the MET currents in *Lhfpl5* knockouts (9), if involving TMC1, would be consistent with the view that the mechanical connection to the tip-link was weakened in the absence of LHFPL5. Without LHFPL5, mechanical stimuli may still be transmitted through a direct link from PCDH15 to the channel (10, 50).

### The Transduction Channel Complex.

Current evidence, derived mostly from coimmunoprecipitation experiments and yeast-two-hybrid screens, suggests that PCDH15, TMC1, TMIE, and LHFPL5 interact to form the transduction complex at the tips of the shorter stereocilia in the mammalian cochlea. The intracellular face of the complex may also be tethered via CIB2 to the stereociliary cytoskeleton (51). Both TMC1 and TMC2 are coimmunoprecipitated by PCDH15 when expressed in HEK cells (10), and PCDH15 has been shown to bind TMC1 and zebrafish TMC orthologs (50). LHFPL5 interacts with PCDH15 (9) and is essential for targeting TMC1 to the stereociliary tips in mice (10). In both biochemical and structural studies, the TM and cytoplasmic domains of PCDH15 have been shown to bind LHFPL5 (9, 52). Together, these observations support the hypothesis that tension in the tip-link is normally transmitted to the channel largely through the interaction of PCDH15 with LHFPL5, which itself is then coupled to TMC1 (40). However, the presence of LHFPL5 is not the only means of force transmission as there are direct connections between PCDH15 and TMC1 (10, 50).

## Discussion

TMC1 is the ion-conducting subunit that forms the core of the multimolecular complex of the MET channel in adult cochlear hair cells. This hypothesis is strongly endorsed by several lines of evidence: 1) over 60 missense or deletion mutations in the *TMC1* gene cause deafness in humans, yet no *TMC2* mutations affect hearing (53); 2) *Tmc1* missense mutations in mice can alter the unitary conductance and Ca^2+^ permeability of the MET channel (4, 7) ; 3) modeling the TMC1 structure based on homology to the TMEM16A chloride channel indicates the presence of an ion-conducting pore between TM4 and TM7 (7, 20) and cryo-EM of the nematode TMC1 complex has also revealed an ion-conducting pore (19); 4) the TMC1 molecule is located at the transduction site in the shorter stereocilia (22, 54); and 5) truncated turtle TMC1 protein has been incorporated into liposomes and shown to produce pressure-activated cationic currents (55).

In considering the structure and delivery of force to the channel complex, it would be helpful to know how many MET channels and TMC1 molecules are present at the transduction site. Hair bundles of mouse apical OHCs contain between 68 and 73 stereocilia (mean = 71 ± 7, *n* = 19), which are denoted as being components of the transducing staircase (56). Since OHC bundles contain three rows of stereocilia, and MET channels are present at the lower ends of the tip-links (3), only two-thirds of the stereocilia contain MET channels. Thus, it can be predicted there are about 47 transduction sites per bundle. We have obtained a range of single-channel conductance values for *Tmc1^+/+^;Tmc2^−/−^* MET channels in apical OHCs, most between 70 and 85 pS (4, 22, 41, 57) and for the following calculation will take an average of 80 pS, equivalent to a current of −6.7 pA at −84 mV. For a maximum OHC MET current of 1,100 pA (Fig. 3*H*) and a single-channel current of 6.7 pA, the number of channels per stereocilium is 1,100/(47 × 6.7) = 3.5. Based on the numbers of stereocilia in OHCs and IHCs at other cochlear locations, similar numbers of channels per tip-link (3.4 to 3.7) can be calculated (Table 1).

**Table 1.** Numbers of stereocilia per bundle and MET channels per tip-link

Hair cell type	*N*_S_, number of stereocilia	*N*_TL_, number of tip-links	*I*_MX_, peak MET current (pA)	MET channels/tip-link
OHC apex (*d* = 0.2)	71 ± 7	47	1,100	3.5
OHC middle (*d* = 0.5)	88 ± 5	58	1,330	3.4
OHC base (*d* = 0.8)	105 ± 6	70	1,720	3.7
IHC apex (*d* = 0.2)	50 ± 5	33	750	3.4
IHC middle (*d* = 0.5)	49 ± 3	33	770	3.5
IHC base (*d* = 0.8)	46 ± 4	31	700	3.5

Number of tip-links, *N*_TL_ = 2 *N*_S_/3, assuming there are three rows of stereocilia per bundle; MET channels/tip-link calculated as *I*_MX_/*N*_TL_/6.7, assuming single-channel current at −84 mV is 6.7 pA throughout. *I*_MX_, peak MET current, is the largest recorded. Stereociliary numbers were derived from counts of scanning electron micrographs of three to nine bundles per location in adult CD1 mice.

**Fig. 6.**
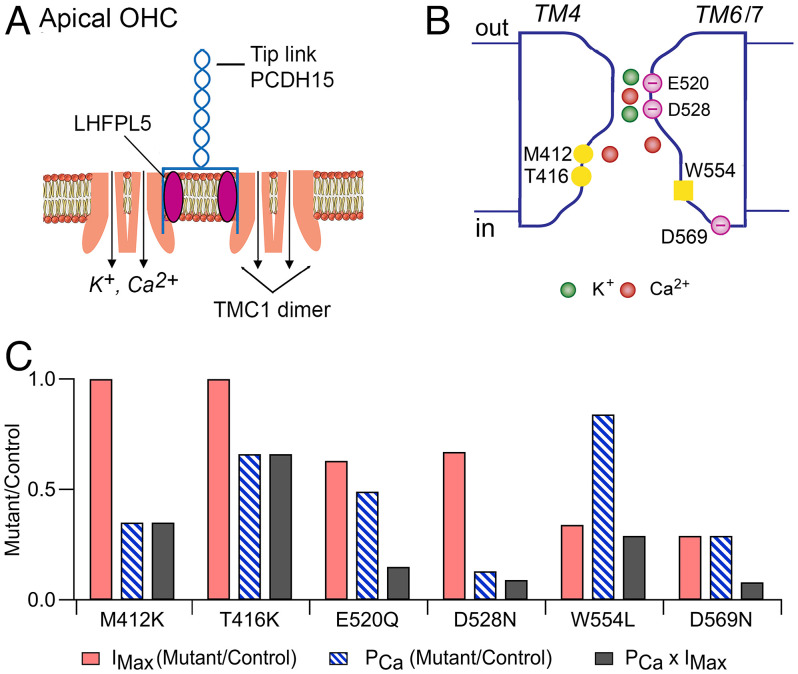
Schematics of OHC transducer complex and TMC1 channel. (*A*) At the cochlear apex, two dimeric TMC1 channels are shown in the stereociliary membrane. Each dimer is attached to an LHFPL5 molecule, which in turn is connected to one PCDH15 of the tip-link. Pore locations indicated by arrows, each transporting K^+^ and Ca^2+^. Not shown are TMIE subunits thought to be linked to TMC1 channel or the CIB2, which is assumed to attach on the inner face of the TMC1. (*B*) Hypothetical cross-section of the channel formed by TM domains 4, 5, 6, and 7, exhibiting an outer vestibule, a neck region (E520, D528), and a larger inner vestibule (M412, T416). W554 on inner face of TM6 and D569 on cytoplasmic link to TM7 may interact with LHFPL5. Mutations discussed in the text nullify the negative charges (E520Q, D528N, D569N) or add a positive charge (M412K, T416K), to increase the electrostatic barrier for transmission of positively charged cations. (*C*) Effects of six homozygous *Tmc1* mutations on the peak MET current (*I*_MX_), Ca^2+^ permeability of the MET channel (*P*_Ca_), and Ca^2+^ influx into the hair bundle (*I*_MX_ × *P*_Ca_). Both *I*_MX_ and *P*_Ca_ are expressed as mutant/control.

These results suggest that variation in number of stereocilia per OHC bundle is a major factor determining the tonotopic current gradient, whereas the lack of gradient in IHCs is due to the lack of gradient in IHC stereocilia per bundle. The range of channels per tip-link is nearly twice the number of PCDH15 carboxy termini (two per tip-link), suggesting some channels may be stimulated without direct connection to PCDH15. However, if each 6.7-pA channel corresponds to one half of the dimeric complex (7, 19), then four channels could be generated by two dimeric complexes, with each complex being connected to a PCDH15 via LHFPL5 (Fig. 6*A*). The reason all channel densities are slightly less than 4.0 may be due to underestimating the maximum current. The predicted numbers of channels at the transduction site (Table 1) are smaller than those (8–20) determined from bleaching of fluorescent TMC1-GFP in the stereocilia (22), and it is likely that the latter values are overestimates.

These calculations of channel numbers also raise concerns about whether the apparent gradient in channel conductance is minor or artifactual. It is conceivable that for high-frequency OHCs, in which the stereocilia are narrower, multiple channels contribute to the records, resulting in conductances of double or triple the unitary value. A further concern is the exact action of low Ca^2+^ plus BAPTA: if its sole effect is to sever the tip-links, then a minimum of three to four channels should remain, implying BAPTA has some other action, for example on the connection between PCDH15 and LHFPL5. The precise effect of low Ca^2+^ plus BAPTA remains to be clarified, as do the exact channel conductance values to reconcile the differences in the literature and whether the gradient in channel conductance exists. An alternate method of MET channel isolation, such as recording from cell-attached patches on single stereocilia, might resolve the discrepancies.

Our studies of hair cell MET currents in mouse harboring *Tmc1* mutations revealed two mutations, *Tmc1* p.D528N and *Tmc1* p.E520Q, which each produced about a 35% decrease in single MET channel conductance, arguing that they are located around the entrance of the pore (Fig. 6 *B* and *C*). This provides further support for TMC1 forming the ion-channel and suggests the neck of the pore is enclosed. All six *Tmc1* mutations studied reduced the Ca^2+^-permeability of the MET channel and, when compounded with a reduced MET current, decreased Ca^2+^ influx into the hair bundle (Fig. 6*C*). The glutamate and the two aspartate substitutions had the largest effect, reducing influx by nearly an order-of-magnitude through reductions in both channel conductance and Ca^2+^ permeability. Associated with a severely reduced Ca^2+^ influx was an early apoptosis of the cochlear hair cells, especially at the base. For example, by P30, *Tmc1* p.D528N showed substantial loss of OHCs and IHCs (4). In contrast, *Tmc1* p.T416K had less hair cell loss at P30, and *Tmc1* p.W554L had no hair cell loss until P60. Even though hair cells harboring the *Tmc1* mutations exhibited MET currents in the first few postnatal days, all mutations eventually led to loss of transduction and deafness by P30. The decreased Ca^2+^ influx into the hair bundle is likely to be a major causal factor: it can lead to decreased actin polymerization and shortening or regression of the transducing stereocilia (58, 59), as well as altering the shape of the hair bundle. In many of the *Tmc1* mutants, OHC bundles are transformed from a typical “V” pattern to a rounded shape by the onset of hearing at P12 (22, 28). Distortions of bundle profile are particularly evident in *Cib2^−/−^*-lacking MET currents (14, 16). The role of Ca^2+^ in determining hair bundle shape is not yet understood.

## Data Availability

All study data are included in the article and all mouse mutants are available on request.
